# Intercalates of Bi_2_Se_3_ studied *in situ* by time-resolved powder X-ray diffraction and neutron diffraction†

**DOI:** 10.1039/d1dt00960e

**Published:** 2021-08-11

**Authors:** Machteld E. Kamminga, Simon J. Cassidy, Partha P. Jana, Mahmoud Elgaml, Nicola D. Kelly, Simon J. Clarke

**Affiliations:** Department of Chemistry, University of Oxford, Inorganic Chemistry Laboratory South Parks Road Oxford OX1 3QR UK simon.clarke@chem.ox.ac.uk

## Abstract

Intercalation of lithium and ammonia into the layered semiconductor Bi_2_Se_3_ proceeds *via* a hyperextended (by >60%) ammonia-rich intercalate, to eventually produce a layered compound with lithium amide intercalated between the bismuth selenide layers which offers scope for further chemical manipulation.

## Abstract 2

New lithium amide/ammonia intercalates of Bi_2_Se_3_ are revealed using *in situ* X-ray powder diffraction.

## Graphical abstract

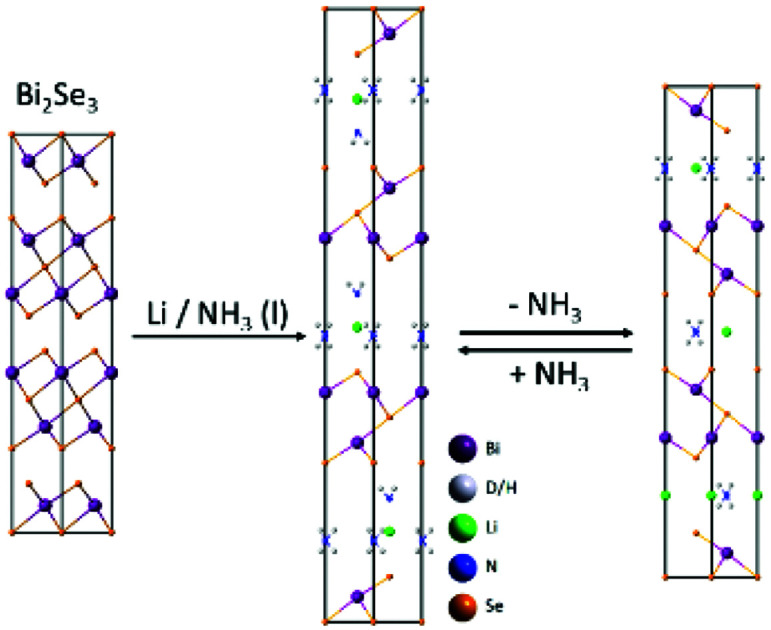

Bismuth selenide, Bi_2_Se_3_, is of contemporary interest as a thermoelectric material,^1–5^ and as a layered topological insulator.^6^ The structure consists of quintuple Se–Bi–Se–Bi–Se layers separated by a van der Waals gap and invites the intercalation chemistry well known in layered chalcogenides.^7–9^ Direct intercalation of zerovalent metals such as Cu into the structure results in complex superstructures;^10^ High temperature reactions of the elements yield Bi_2_Se_3_ derivatives containing Cu, Sr and Nb which exhibit superconductivity.^11–13^ These are often described as intercalates with the assumption that the additional metal occupies the interlayer space, although many of these are not well characterised with respect to either composition or crystal structure. In several layered materials, co-intercalation of alkali metals and ammonia/amide has shown to produce dramatic changes in physical properties, *e.g.* in the case of the superconductor FeSe.^14–16^ Here we probe the reaction between Li/ammonia solutions and Bi_2_Se_3_ to produce two products with different amide/ammonia contents that invite further investigations and expand the scope of this chemistry.

The experimental details of the intercalation reactions and the characterisation methods are described in the ESI.†Fig. 1 shows portions of the synchrotron X-ray diffractograms showing the evolution of the lowest angle 003 Bragg reflection of Bi_2_Se_3_ (at 2*θ* ∼ 5°) and the final intercalated Li_*x*_(NH_2_/NH_3_)_*y*_Bi_2_Se_3_ product obtained in reactions with varying Li : Bi_2_Se_3_ ratios (0.2 ≤ *x* ≤ 1.0). A large shift in the lowest angle Bragg peak to still lower angles is observed, indicating a large increase in the separation of the quintuple layers upon intercalating Li and ammonia into the van der Waals gap of Bi_2_Se_3_. As a control, it was checked that suspending Bi_2_Se_3_ in liquid ammonia without any Li did not result in any changes in the diffraction pattern. For small amounts of Li (*x* = 0.2 and 0.3) not all the Bi_2_Se_3_ was involved in the intercalation reaction, but a new reflection emerged at 2*θ* ∼ 4.5° (*i.e. d* ∼ 10.5 Å). Upon increasing *x* to 0.4, no crystalline Bi_2_Se_3_ remained and a very broad peak was observed with a centre of gravity at 2*θ* ∼ 3.9° (*i.e. d* ∼ 12 Å). Addition of up to one mole of Li per mole of Bi_2_Se_3_ produced a single phase product with a narrow 003 peak at 2*θ* ∼ 3.65° (*i.e. d* ∼ 12.9 Å). Rietveld refinements of Bi_2_Se_3_ and Li_*x*_(NH_2_/NH_3_)_*y*_Bi_2_Se_3_ with *x* = 1.0 are shown in Fig. S1, ESI.† Note that the peak shapes for the intercalated phase are broader than in the parent Bi_2_Se_3_ with the profile parameters suggesting strain-related broadening. The very broad peaks for *x* < 1 suggest stacking disorder, which requires further analysis.

**Fig. 1:**
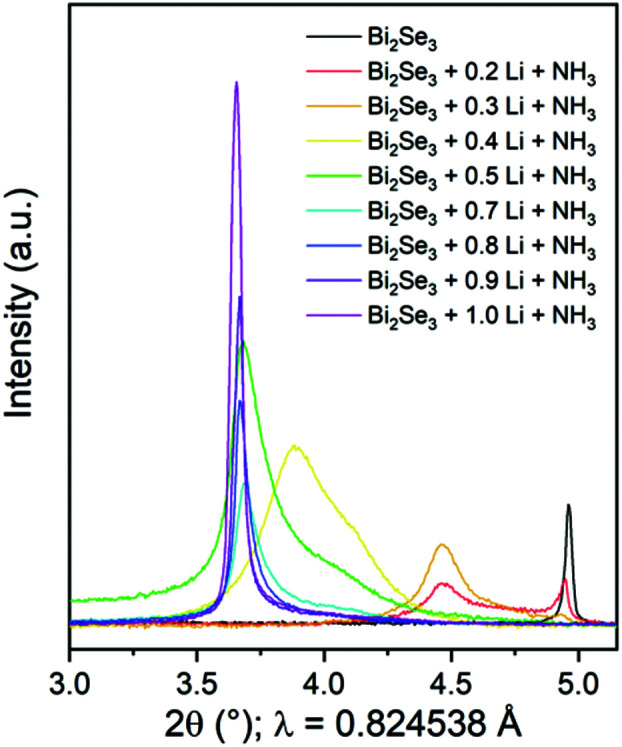
The lowest angle 003 peak of Bi_2_Se_3_ and Li_*x*_(NH_2_/NH_3_)_*y*_Bi_2_Se_3_ with varying targeted Li content (0.2 ≤ *x* ≤ 1.0).

X-ray diffraction intensities are dominated by the contributions from Bi and Se, so powder neutron diffraction (PND) measurements on the crystalline intercalates obtained by reacting Li/NH_3_ with Bi_2_Se_3_ in a Li : Bi_2_Se_3_ ratio of 1 : 1 were conducted. Samples of both Li_*x*_(NH_2_/NH_3_)_*y*_Bi_2_Se_3_ and Li_*x*_(ND_2_/ND_3_)_*y*_Bi_2_Se_3_ were measured at 5 K and room temperature to attempt to constrain the refined models with the expectation of some disorder in the intercalates. H and D have very different neutron scattering lengths (−3.741 fm for H and +6.671 fm for D), H is a strong incoherent scatterer, and H and Li both have negative scattering lengths. Selected diffraction patterns and Rietveld refinements are given in Fig. 2 and Fig. S2, ESI.† A single structural model was refined against all four datasets as explained further in the ESI.†

**Fig. 2:**
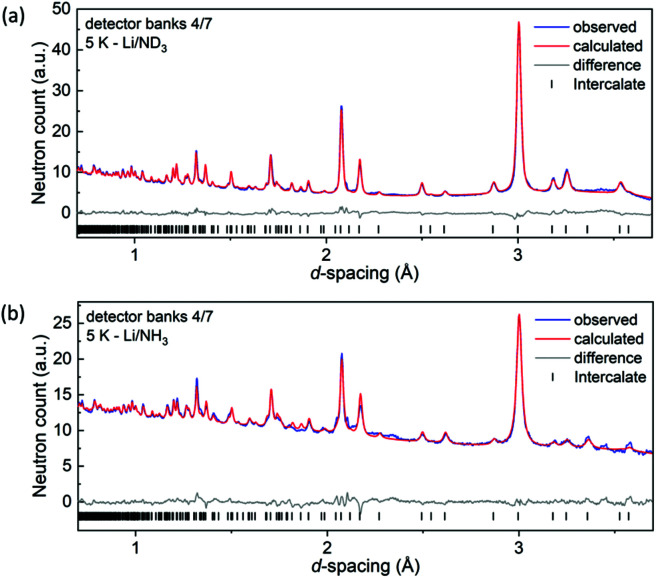
Rietveld refinements of the structure of the (a) Li/ND_3_ and (b) Li/NH_3_ intercalates against POLARIS data at 5 K. Peak positions are marked by vertical lines.

Structural and refinement parameters are listed in Table S1, ESI.†

A positive scattering centre located at (1/3, 2/3, 0.50020(6)) – a trigonal prismatic site formed by Se atoms of adjacent Bi_2_Se_3_ layers – corresponded to an occupancy of 0.44(1) N. There was no sign of a net negative scattering centre corresponding to Li. Taking the different scattering lengths into account (+9.36 fm for N and −1.90 fm for Li), the scattering centre at (1/3, 2/3, 0.50020(6)) corresponds to ∼0.53(1) N and ∼0.47(1) Li, suggesting disorder of Li and NH_2_/NH_3_ moieties on the length scale probed by diffraction and with *x* = *y* = 1 in the formula Li_*x*_(NH_3_/NH_2_)_*y*_Bi_2_Se_3_, consistent with the Li : Bi_2_Se_3_ ratio in the synthesis. A realistic structural model for a single intercalate layer (Fig. S3, ESI†), has each NH_*x*_ moiety surrounded strictly by three Li (and *vice versa*) at ∼2.4 Å comparable to the Li–N bond distance of ∼2.0–2.2 Å crystalline LiNH_2_.^17^ In our model these intercalate layers are disordered along the *c*-direction, hence the average scattering at the (1/3, 2/3, 0.50020(6)) site corresponds to the Li/N disorder on the lengthscale probed by the diffraction experiment. The H and D atoms were located approximately 1 Å from the N atoms, while applying a soft restraint for the N–H/D distance, and this results in weak N–H⋯Se hydrogen bonds of ∼2.8 Å (H⋯Se distance), similar to ammonia intercalates of FeSe^14^ and TiS_2_.^18^ In this model the amide moieties are orientationally disordered on the lengthscale of the diffraction experiment. No further scattering density corresponding to further NH_2_ or NH_3_ moieties could be located. The refinement yielded an H (or D) to N ratio of 2.10(2), so within the experimental uncertainty the intercalate layer is neutral lithium amide and the overall formula is LiNH_2_Bi_2_Se_3_. Chemical analysis (Elemental Microanalysis Ltd, Okehampton, Devon, UK: CHN using the Dumas combustion method) of three samples, including the H-and D-containing samples used in the neutron diffraction experiment yielded a composition LiN_0.9(1)_H_2.1(1)_Bi_2_Se_3_, consistent with the neutron analysis. Consistent with this, SQUID magnetometry (Fig. S4, ESI†) shows a minimal change in the overall diamagnetic susceptibility (from −3.19(6) × 10^−4^ to −2.72(9) × 10^−4^) with no evidence for a substantial injection of electrons into the conduction band to produce a Pauli paramagnetic susceptibility to oppose the diamagnetism of the core electrons. This is consistent with the difficulty of partially reducing Bi^3+^. Conductivity measurements were hampered by the air sensitivity and thermal sensitivity (see below) of these finely divided powders.

To probe the course of the intercalation reaction, we performed the reaction *in situ* at the I12 beamline at the Diamond Light Source (see ESI† for details). Fig. 3(a) shows the diffraction patterns measured at four different time stamps. The red pattern shows the background (see ESI†). The blue pattern (*t* = 0 s) shows the synchrotron PXRD (powder X-ray diffraction) pattern directly after tipping the Bi_2_Se_3_ into the Li/NH_3_ solution, which corresponds to pure Bi_2_Se_3_, (Fig. S6(a), ESI†). The characteristic first peak with a *d*-spacing of 9.483(2) Å (003 reflection in the hexagonal setting of space group *R*3̄*m*) corresponds to the separation between adjacent Bi_2_Se_3_ quintuple layers. After about two minutes, a second set of diffraction peaks appears, with the first reflection at 15.380(3) Å, indicating that the interlayer distance has increased by a remarkable 62% upon intercalation. Fig. 3(b and c) show the detailed time lapse of the intercalation.

**Fig. 3:**
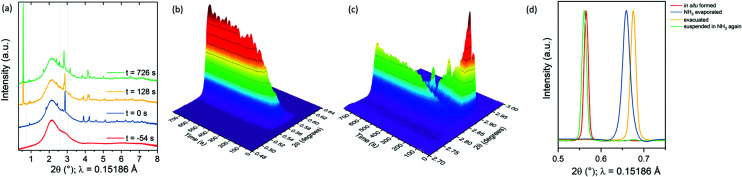
(a) Diffraction patterns from integration of the Bragg rings measured on I12 at four different moments during the *in situ* reaction. The patterns are off-set for clarity. The background from the glass and liquid was treated as described in the ESI.† The dotted lines indicate the ranges plotted in (b) and (c). Time lapse of the *in situ* PXRD measurement showing (b) the rise of the 003 peak of the intercalate phase and (c) the simultaneous decrease of the 015 peak of the parent compound and the rise of the 018 peak of the intercalate phase over time. (d) 003 peak of the ammonia-rich intercalate as formed during the *in situ* reaction, after evaporation of NH_3_, after evacuation, and after suspending again in liquid ammonia, showing the reversible nature of ammonia desorption as shown in Fig. 4. The background is subtracted for clarity.

The product obtained in this *in situ* experiment is measured while suspended in liquid ammonia and has a much larger interlayer separation than the dry product described above using NPD. After boiling off the ammonia and evacuating the reaction vessel to produce a dry product as in the lab experiment, the first peak shifts to 12.83(1) Å corresponding to the lab-synthesised product LiNH_2_Bi_2_Se_3_ described in detail above with an interlayer distance 35% larger than in Bi_2_Se_3._ Note that evacuation is necessary to fully develop the ammonia-poor compound (see Table 1). The highly expanded phase can be regained by suspending the dry evacuated product in liquid ammonia shown in Fig. 3(d) and Fig. S5, ESI,† suggesting that the new phase identified in the *in situ* measurement is an ammonia rich phase. Fig. 3(d) shows that evacuation is required to fully remove all the NH_3_ molecules from the initial intercalate to result in the product LiNH_2_Bi_2_Se_3_. We note that suspending the evacuated product in liquid ammonia gives rise to a re-ammoniated product with a very slightly different *d*_003_-spacing to that of the initial intercalate measured *in situ* (by less than 1%). Because the sample was removed from the diffractometer and placed back into the beam between each step in Fig. 3(d), we cannot rule out that this small difference is an artefact of the experiment, although the two ammonia-rich phases were obtained by different routes, and may differ slightly in their level of intercalated ammonia.

**Table 1:** Lattice parameters and cell volumes of Bi_2_Se_3_ and intercalates, determined from I12 PXRD data. The colour coding corresponds to Fig. 3(d) and the bold-faced data corresponds to the structures drawn in Fig. 4

		*a* (Å)	*c* (Å)	*V* (Å^3^)	*d*_003_-spacing = *c*/3 (Å)
	**Bi**_**2**_ **Se**_**3**_	**4.1157(4)**	**28.449(4)**	**417.3(1)**	**9**.**483(2)**
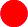	**Ammonia rich intercalate**	**4.1852(3)**	**46.140(7)**	**699.91(14)**	**15.380(3)**
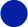	Evaporated intercalate	4.1928(9)	39.68(3)	604.1(5)	13.23(1)
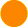	**Evacuated intercalate**	**4.1780(11)**	**38.49(3)**	**581.88(5)**	**12.83(1)**
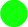	Suspended again intercalate	4.1634(4)	46.467(11)	697.54(19)	15.489(4)

The superconducting Li/NH_3_ intercalates of FeSe also show ammonia-rich and ammonia-poor phases. In that case the ammonia-rich phase could be stabilised in dry form by exposing it to 1 bar of ammonia gas at −20 °C.^15^ The ammonia-rich intercalated phase of Bi_2_Se_3_ could not be regenerated in the dry form, hampering analysis by NPD. The I12 synchrotron data (Rietveld refinements shown in Fig. S6, ESI†) together with sensible assumptions about bond lengths was used to propose a model for the crystal structure as shown in Fig. 4(b). Fig. S7, ESI,† shows that adding one mole of ammonia per mole of LiNH_2_Bi_2_Se_3_ results in a tetrahedral coordination of N around Li with sensible interatomic distances: the Li–NH_2_ distance is ∼2.5 Å and the N–H⋯Se hydrogen bonds have H⋯Se ∼2.9 Å, indicating weak hydrogen bonds, which is consistent with the distance found in other intercalates.^14,18^

**Fig. 4:**
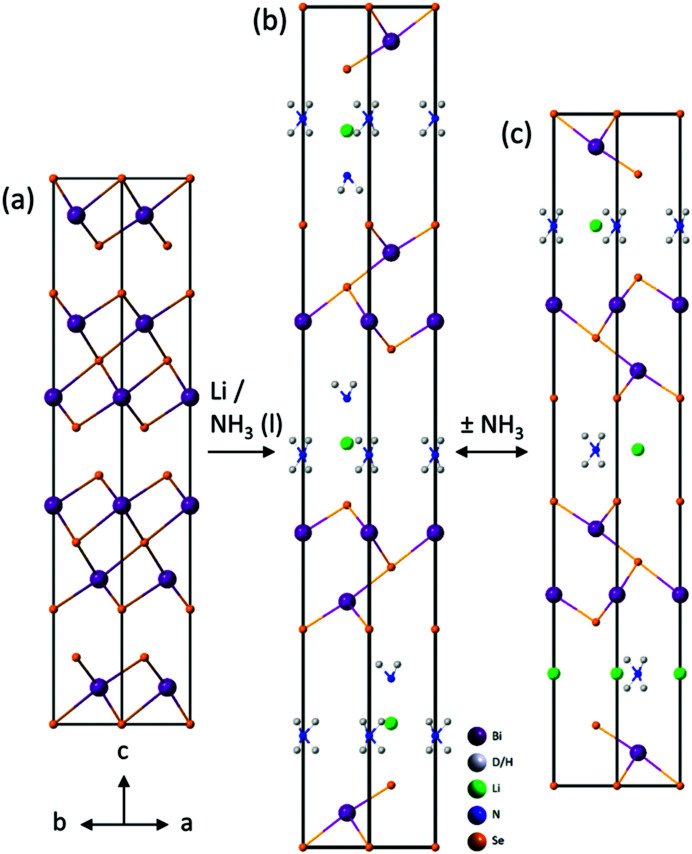
(a) Crystal structure of Bi_2_Se_3_, (b) proposed model for ammonia-rich intercalate, and (c) model from Rietveld refinement of the Li-amide-intercalated LiNH_2_Bi_2_Se_3_. Note that in (b) and (c), Li and N are disordered on the lengthscale probed by XRD and possible arrangements are shown for clarity.

Based on the abovementioned model for the intercalated end product as determined by neutron diffraction, we extended the model to incorporate our suggested intermediate phase to: LiNH_2_(NH_3_)_*z*_Bi_2_Se_3_, where *z* ≤ 1 NH_3_ can be added or removed by changing between the final and intermediate phase (see Fig. 4). The lattice parameters are given in Table 1.

In Bi_2_Se_3_ the quintuple layers are stacked in an ABCABC-type fashion, resulting in the rhombohedral symmetry. Upon intercalation to form the initial ammonia-rich phase, this stacking is maintained. Upon drying, with the loss of the ammonia to form LiNH_2_Bi_2_Se_3_, a rearrangement of the layers occurs resulting in an ACBACB-type stacking. This maintains rhombohedral symmetry, but has a different relative arrangement of the layers (Fig. 4(c)) with the layers of selenide ions coordinated to the intercalated species eclipsed when viewed along the *c* direction, while in Bi_2_Se_3_ and in the ammonia-rich intercalate phase they are staggered (Fig. 4(a and b)). These changes are presumably driven by the coordination requirements of the intercalated molecules, and these changes mimic those that are found in the Li/NH_3_ intercalates of FeSe.^14,15^

LiNH_2_Bi_2_Se_3_ decomposes on heating above 450 K. As shown in Fig. S8, ESI,† between 480 and 490 K there is a broadening of the lowest angle reflection, together with a dramatic shift with a new, fairly crystalline phase formed at 490 K which has the first reflection equated with the interlayer separation at ∼11.8 Å, smaller than in the intercalate phases, but much larger than in Bi_2_Se_3_. The relatively broad diffraction peaks of this phase hampered further structural characterisation, but it suggests further complexity in the intercalated Bi_2_Se_3_ phase field. Heating above 495 K resulted in further decomposition.

These results show that in intercalates of layered compounds with metal ions and small molecules the adoption of both molecule-rich and molecule-poor structures is not uncommon. These intercalates do not show desirable properties such as the superconductivity reportedly induced in other derivatives of Bi_2_Se_3_, but they offer a starting point for further compositional tuning to tune electronic properties, and chemical routes to new materials *via* exfoliation and ion exchange. Further investigations of Li and H/D mobilities and the range of compositions available in the ammonia-rich and ammonia-poor phase fields are in progress.

## Author contributions

MEK, PPJ, SJCa, ME and NDK performed the chemical synthesis. MEK and SJCa prepared samples for neutron powder diffraction and analysed these and the other characterisation data with input from SJCl. MEK and SJCl wrote the paper with input from the other authors.

## Conflicts of interest

There are no conflicts to declare.

## Supplementary Material

DT-050-D1DT00960E-s001
