# Supplementary material for: Intercalates of Bi2Se3 studied in situ by time-resolved powder X-ray diffraction and neutron diffraction
Source: Dalton Trans. 2021 Aug 11;50(33):11376–9. doi: 10.1039/d1dt00960e (PMC8383151; doi:10.1039/d1dt00960e)
Supplement: DT-050-D1DT00960E-s001 [file DT-050-D1DT00960E-s001.pdf]

Electronic Supplementary Material (ESI) for Dalton Transactions.
This journal is © The Royal Society of Chemistry 2021

Electronic Supplementary Information (ESI) for

Intercalates of Bi_2Se_3 Studied *in Situ* by Time-Resolved Powder X-ray Diffraction and Neutron Diffraction

Machteld E. Kamminga,[a] Simon J. Cassidy, Partha P. Jana,[b] Mahmoud Elgaml, Nicola D. Kelly,[c] Simon J. Clarke[*]

Department of Chemistry, University of Oxford, Inorganic Chemistry Laboratory, South Parks Road, Oxford OX1 3QR, United Kingdom.

[a] Current address: *Niels Bohr Institute, University of Copenhagen, Universitetsparken 5, DK-2100 Copenhagen Ø, Denmark.*

[b] Current address: *Department of Chemistry, IIT Kharagpur, India-721302.*

[c] Current address: *Cavendish Laboratory, Department of Physics, University of Cambridge, JJ Thomson Avenue, Cambridge CB3 0HE, United Kingdom.*

[*] E-mail: simon.clarke@chem.ox.ac.uk

Experimental Section

Synthesis

All manipulations were carried out in a Glovebox Technology argon-filled dry box with an O_2 and H_2O content below 1 ppm or on a Schlenk line. The parent compound Bi_2Se_3 was synthesized by mixing together high-purity Bi pieces (Sigma Aldrich; 99.999%) and Se powder (Alfa Aeser; 99.999%) and heating them in a sealed silica tube to 720 °C at 2 °C min^{-1}, holding the temperature for 48 h, cooling to room temperature at the natural rate of the furnace, grinding the product into a fine powder and repeating the heating procedure with the same ramp rate and holding at 720 °C for 72 h before cooling to room temperature.

The intercalates, $Li_x(NH_2/NH_3)_yBi_2Se_3$ and $Li_x(ND_2/ND_3)_yBi_2Se_3$ with targeted compositions $0.2 \leq x \leq 1.0$, were synthesised by placing the appropriate stoichiometric quantities of Li metal (Sigma Aldrich; 99%) and Bi_2Se_3 in a Schlenk tube with a magnetic stirrer bar. The Schlenk tube and a cylinder of ammonia (NH_3: BOC; 99.98 %, or ND_3: Sigma Aldrich; 99% D) were attached to a Schlenk line. After evacuating all pipework between the ammonia cylinder and the sample space, the Schlenk tube was placed in a slush bath of dry ice and isopropanol (-78 °C) to allow condensation of ammonia onto the reactants. For 0.40 g of Bi_2Se_3, around 15 mL of ammonia was condensed into the Schlenk tube. The solution was observed to briefly turn blue, characteristic of solvated electrons, after which the solution decoloured. The Schlenk tube was allowed to warm with the slush bath to room temperature over the course of around 4 h while boiling off ammonia [safety note: ammonia is volatile and toxic. Therefore, the Schlenk line was constructed so that any ammonia pressure could be relieved at all times via a mercury bubbler]. After evacuating the Schlenk tube, a dry dark grey powder was obtained.

Powder neutron diffraction

Powder neutron diffraction (PND) experiments were performed at 5 K and room temperature on the POLARIS diffractometer[1] at the ISIS Facility, Rutherford Appleton Laboratory, UK. Samples with NH_3 or ND_3 were contained in 6-mm-diameter thin-walled vanadium cans that were sealed with indium gaskets. Rietveld analysis was performed with TOPAS-Academic V5.[2]

In situ X-ray diffraction

The synthesis of the ammonia-rich Li-intercalated Bi_2Se_3 were performed on the I12 beamline[3] at the Diamond Light Source (UK) in an experiment conceived to investigate the structural changes during the intercalation process *in situ* and identify possible intermediate phases. The set-up was similar to that described previously for the intercalates of FeSe.[4] In an argon-filled glovebox, 3.2 (0.461 mmol) mg of Li and a small magnetic stirrer bar were loaded into the bottom of a 18 mm o.d × 4 mm wall Pyrex ampules sealed with high pressure Rotaflow Teflon valve [safety note: since these ampules were to be sealed with liquid ammonia at room temperature or below, they were professionally constructed and tested to withstand a pressure of 15 bar]. A stoichiometric amount of freshly synthesized Bi_2Se_3 (0.30 g, 0.461 mmol) was carefully placed into a side arm of the ampule to prevent direct contact with the Li. A Schlenk line setup was used to condense around 5 mL of ammonia onto the Li by placing the bottom of the ampule containing Li in a liquid nitrogen bath. On melting the ammonia ice in a -78 °C slush bath (mixture of dry ice and isopropanol), the Li fully dissolved and a blue solution characteristic of solvated electrons was obtained. The ampule was sealed and carefully transported to the beamline, with its bottom half in a bucket filled with slush, while maintaining the Bi_2Se_3 out of contact with the Li/NH_3 solution. The ampule was placed with the solution in the beam while clamped to a remote-controlled rotational stage and kept cool with a cold argon flow. While stirring the Li/NH_3 solution, diffractograms were measured upon exposure of the monochromatic 81.6 keV (λ = 0.15186 Å) X-ray beam and collected using a Pilatus CdTe 2M detector. Frames were collected continuously in time intervals of 100 ms separated by 400 ms dead time. To initiate the reaction, the ampule was rotated about the beam direction so that the Bi_2Se_3 poured out of the side-arm and into the solution. The evolution of the diffraction pattern was monitored for a few minutes to a few hours, depending on the reaction temperature used. Data reduction to integrate the diffraction pattern recorded on the 2D detector was performed using DAWN.[5] Data analysis of the powder data reduced to one dimension was performed with TOPAS-Academic V5.[2] Additional powder diffraction experiments on the intercalates, $Li_x(NH_2/NH_3)_yBi_2Se_3$, synthesised in-house with targeted compositions $0.2 \leq x \leq 1.0$ were performed on the I11 beamline[6] at the Diamond Light Source (UK) using 0.82 Å X-rays.

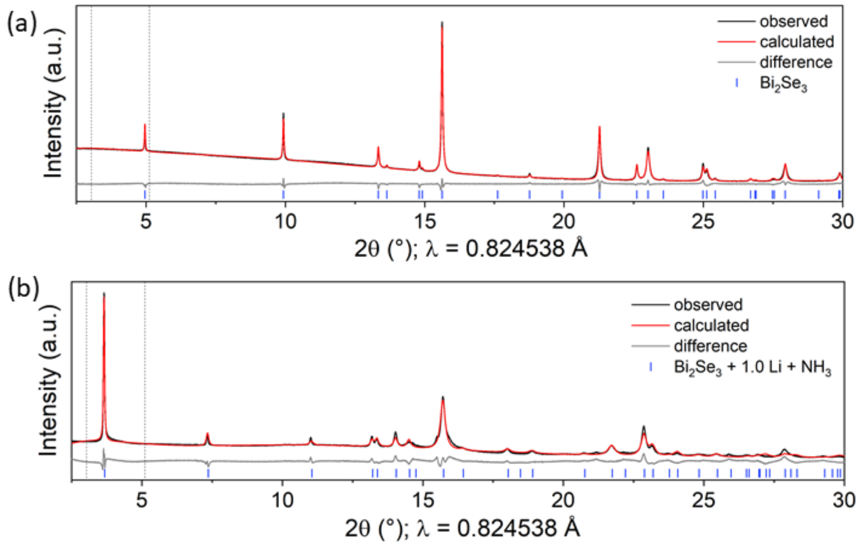

Fig. S1 Rietveld fits to I11 synchrotron data of Bi_2Se_3 and $Li_x(NH_2/NH_3)_yBi_2Se_3$ with $x = 1.0$, respectively. The dotted lines indicate the range plotted in Fig 1. The data in **(b)** is refined against Bi and Se positions only, as these heavy elements dominate the X-ray pattern (see main text).

Description of Rietveld refinements

A coupled Rietveld refinement for the highest resolution banks 3/8, 4/7 and 5/6 of the POLARIS diffractometers was performed on all four data sets in which a single structural model was used to fil all patterns. In this refinement, the lattice parameters for each data set were refined independently, to allow for small differences between the H- and D-containing samples and the changes in temperature, while the atomic positions and occupancies were coupled between each data set (*i.e.* the assumption was made that the structural model was temperature independent). Moreover, all atoms were refined with identical isotropic displacement parameters: one for the 5K data sets and one for the room temperature data sets. By assuming that substituting D for H does not impact the intercalations, and by benefitting from their different neutron scattering lengths, we forced the H and D occupancies to be the same in order to completely model the intercalated structure.

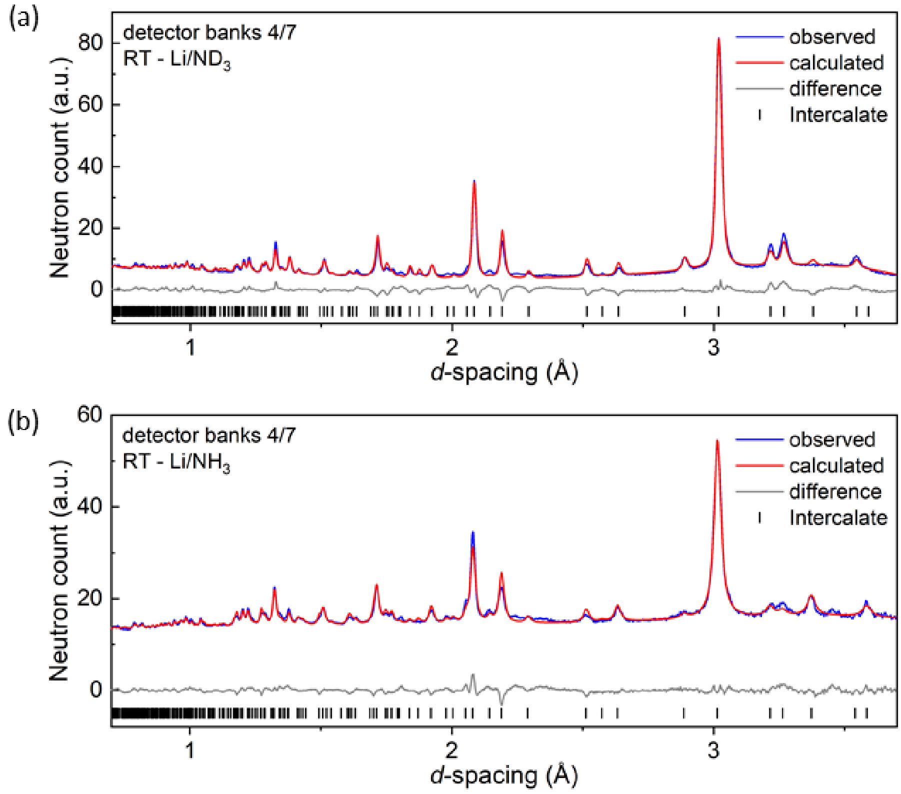

Fig. S2 Observed (blue) and calculated (red) powder neutron diffraction patterns and difference profile (grey) of the Rietveld refinements of the structure of the ammonia-poor **(a)** Li/ND_3 and **(b)** Li/NH_3 intercalates against POLARIS data at room temperature. Peak positions are marked by vertical lines.

Table S1. Structural and refinement parameters of POLARIS neutron diffraction data (esd's in parentheses).

Crystal Structure Data				
formula	$LiNX_2Bi_2Se_3$ (X = D/H)			
crystal system	Trigonal			
space group	*R*-3*m*:*H*			
formula units *Z* / cell	3			
temperature	5 K	5 K	room temperature	room temperature
ammonia	ND_3	NH_3	ND_3	NH_3
cell parameters / Å	*a* = 4.1484(4)	*a* = 4.1388(7)	*a* = 4.1657(5)	*a* = 4.1582(9)
	c = 38.136(4)	*c* = 38.092(7)	*c* = 38.598(5)	*c* = 38.585(9)
cell volume / Å^3	568.36 (13)	565.1(3)	580.06(16)	577.8(3)
Data collection				
diffractometer / radiation	POLARIS neutron diffractometer, time-of-flight measurement			
d-spacing range bank 1 / Å	0.94280 - 40.24778			
d-spacing range bank 2 / Å	0.66932 - 13.87116			
d-spacing range bank 3 / Å	0.32659 - 5.91452			
d-spacing range bank 4 / Å	0.20384 - 3.69421			
d-spacing range bank 5 / Å	0.14893 - 2.69895			
Structure Refinement				
method of refinement	Rietveld			
software	TOPAS-Academic V5			
r_wp refinement parameter	3.476			

Structure Parameters[a]			
Atom	Site	occupancy	coordinates *x*, *y*, *z*
Bi	*6c*	1	0, 0, 0.28274(3)
Se1	*6c*	1	0, 0, 0.42272(3)
Se2	*3a*	1	0, 0, 0
Li / N1	*6c*	0.5	1/3, 2/3, 0.50020(6)
X1	*18h*	0.166(3)	0.2510(8), -0.2510(8), 0.51942(11)
X2	*18h*	0.184(3)	0.2512(6), -0.2512(6), 0.4844(3)

[a]Refinement of atomic positions and occupancies were coupled for all four phases.

[b]A single isotropic displacement parameter was used for all atoms at each temperature: 0.0049(2) Å^2 at 5 K; 0.021(1) Å^2 at RT

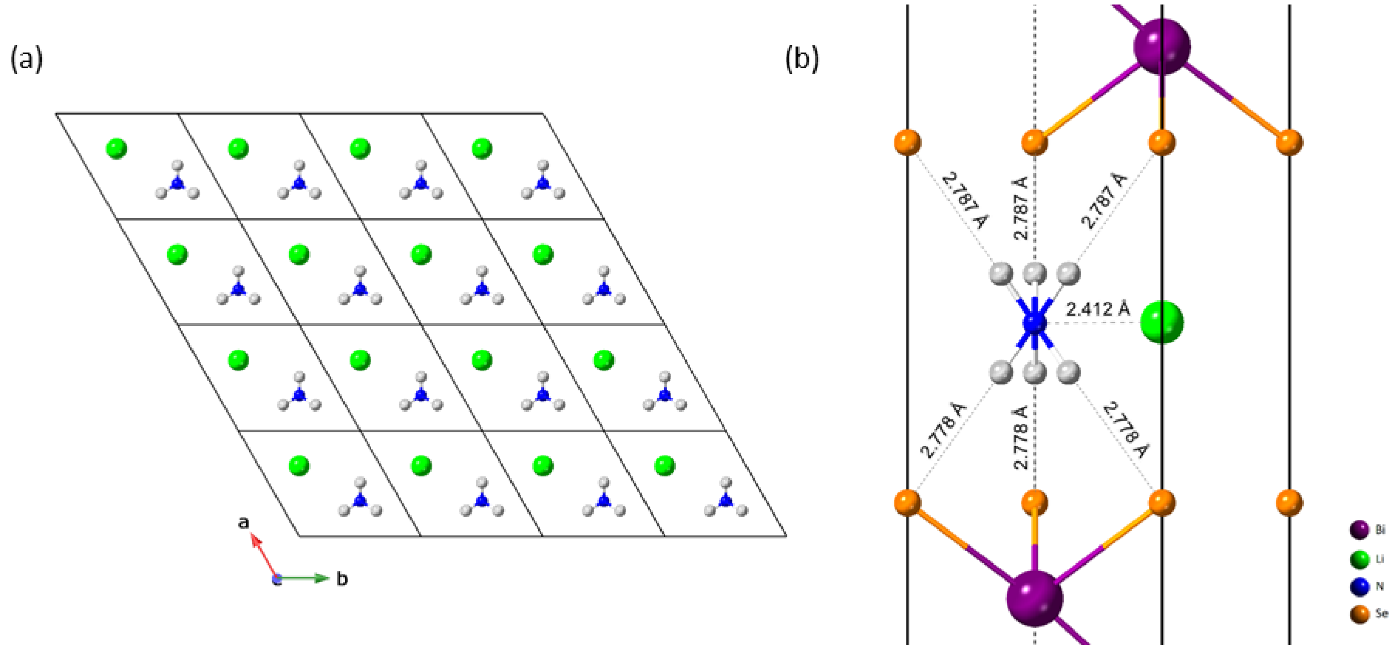

Fig. S3 (a) A single slab of Li and amide as intercalated in the van der Waals gap present in Bi_2Se_3, showing the honeycomb type lattice formed by the intercalates. In our model each layer is ordered, but there is stacking disorder of the intercalate layers leading to the average structure probed by diffraction having Li and NH_2^- moieties sharing a single crystallographic site. Note that the amide is orientationally disordered. **(b)** $LiNH_2Bi_2Se_3$ intercalate projected along the [210] direction, showing interatomic distances from the Rietveld refinement (Table S1).

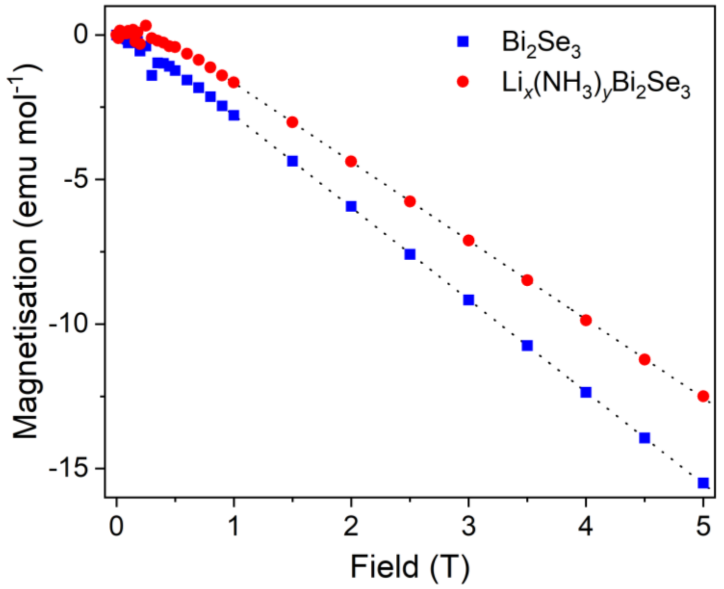

Fig. S4 *M* vs *H* for Bi_2Se_3 (blue) and its Li and ammonia intercalate (red) at 300 K. A magnetic impurity was evident at low fields but saturated above 0.5 T. The high-field linear fits (dashed lines) show that both phases are net diamagnets. Calculation of the magnetic susceptibility from the slope of the linear fits gives values of $-3.19(6) \cdot 10^{-4}$ and $-2.72(9) \cdot 10^{-4}$ for Bi_2Se_3 and its Li and ammonia intercalate, respectively.

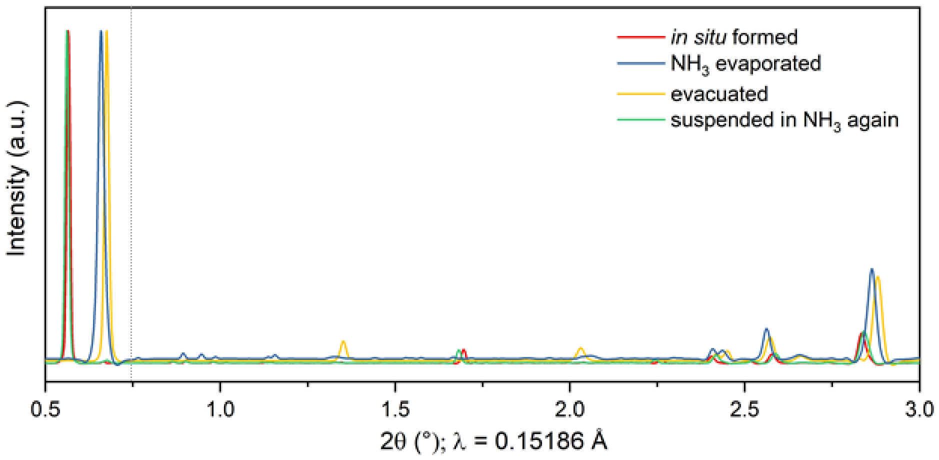

Fig. S5 PXRD pattern of the ammonia-rich intercalate as formed during the *in situ* reaction, after evaporation of NH_3, after evacuation, and after suspending again in liquid ammonia, showing the reversible nature of ammonia desorption as shown in Fig. 5. The background is subtracted for clarity. The dotted framework corresponds to the range plotted in Fig. 4. The lattice parameters of each structure is given in Table 1.

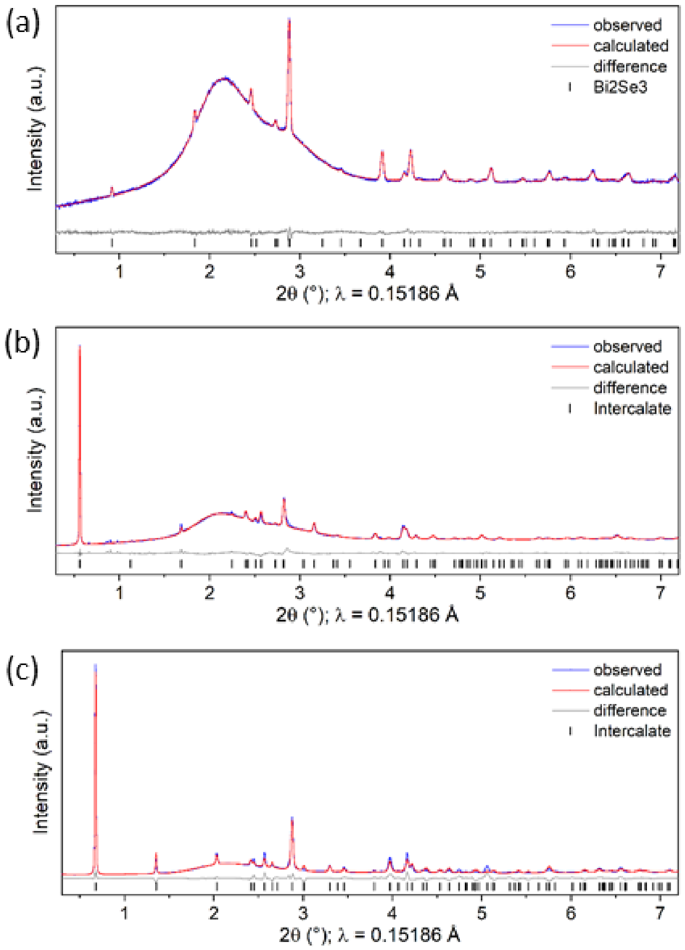

Fig. S6 Rietveld refinements against **(a)** the starting compound Bi_2Se_3, **(b)** the ammonia-rich intercalate phase and **(c)** the dried and evacuated final intercalate product, measured on the I12 beamline at the Diamond Light Source Ltd in the UK. Peak positions are marked by vertical lines. Corresponding schematic of crystal structures are given in Fig. 5.

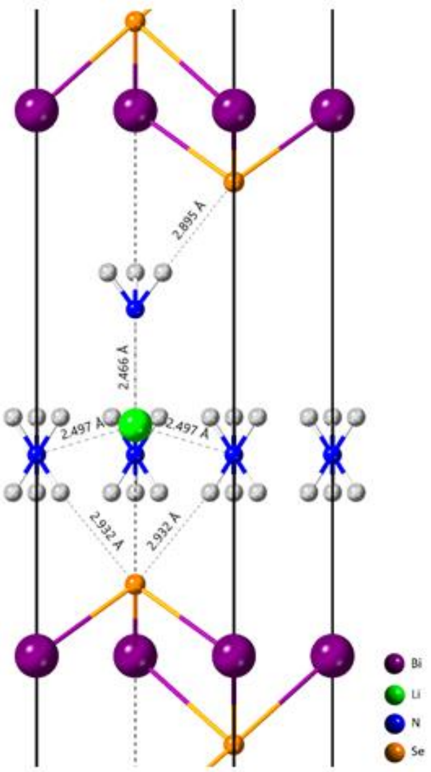

Fig. S7 Proposed model for the ammonia-rich intercalate projected along the [210] direction, showing interatomic distances.

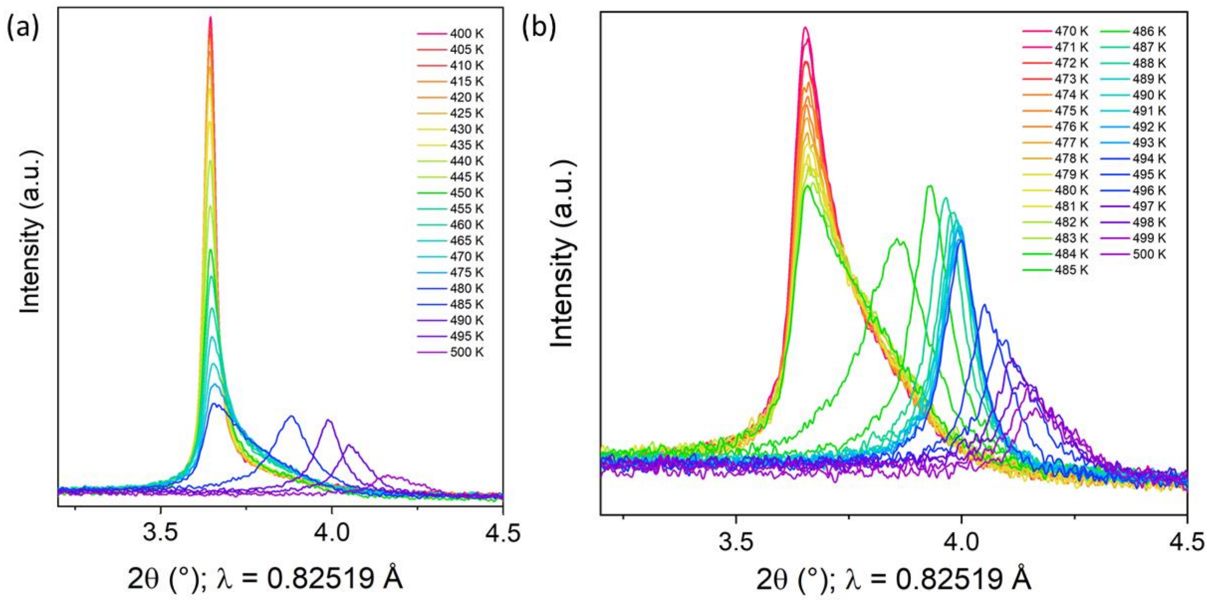

Fig. S8 (a) The 003 peak of $Li_2NH_2Bi_2Se_3$ as a function of temperature between 400 and 500 K, measured *in situ*. **(b)** The same 003 peak as a function of temperature between 470 and 500 K indicating the formation of an additional crystalline phase with a *d*-spacing of ~ 11.8 Å formed by annealing, prior to decomposition at elevated temperatures.

References

1 R. I. Smith, S. Hull, M. G. Tucker, H. Y. Playford, D. J. McPhail, S. P. Waller and S. T. Norberg, *Rev. Sci. Instrum.*, 2019, **90**, 115101.

2 A. A. Coelho, *J. Appl. Cryst.*, 2018, **51**, 210–218.

3 M. Drakopoulos, T. Connolley, C. Reinhard, R. Atwood, O. Magdysyuk, N. Vo, M. Hart, L. Connor, B. Humphreys, G. Howell, S. Davies, T. Hill, G. Wilkin, U. Pedersen, A. Foster, N. De Maio, M. Basham, F. Yuan and K. Wanelik, *J. Synchrotron Radiat.*, 2015, **22**, 828–838.

4 S. J. Sedlmaier, S. J. Cassidy, R. G. Morris, M. Drakopoulos, C. Reinhard, S. J. Moorhouse, D. O'Hare, P. Manuel, D. Khalyavin and S. J. Clarke, *J. Am. Chem. Soc.*, 2014, **136**, 630–633.

5 M. Basham, J. Filik, M. T. Wharmby, P. C. Y. Chang, B. El Kassaby, M. Gerring, J. Aishima, K. Levik, B. C. A. Pulford, I. Sikharulidze, D. Sneddon, M. Webber, S. S. Dhesi, F. Maccherozzi, O. Svensson, S. Brockhauser, G. Náray and A. W. Ashton, *J. Synchrotron Radiat.*, 2015, **22**, 853–858.

6 C. A. Murray, J. Potter, S. J. Day, A. R. Baker, S. P. Thompson, J. Kelly, C. G. Morris, S. Yang and C. C. Tang, *J. Appl. Cryst.*, 2017, **50**, 172–183.
